# Effect of High CO_2_ Controlled Atmosphere Storage on Postharvest Quality of Button Mushroom (*Agaricus bisporus*)

**DOI:** 10.3390/foods13213486

**Published:** 2024-10-30

**Authors:** Yuxian Yang, Ouyang Jia, Yunzhi Li, Bing Feng, Mingchang Chang, Junlong Meng, Bing Deng

**Affiliations:** 1 College of Food Science and Engineering, Shanxi Agricultural University, Jinzhong 030801, China; soph0731@163.com (Y.Y.); m19834542144@163.com (O.J.); 18214659894@163.com (Y.L.); 15738227612@163.com (B.F.); sxndcmc@163.com (M.C.); 2 Shanxi Key Laboratory of Edible Fungi for Loess Plateau, Jinzhong 030801, China

**Keywords:** *Agaricus bisporus*, controlled atmosphere, enzymatic browning, senescence, gene expression

## Abstract

The *Agaricus bisporus* (Button mushroom) stands out as one of the most prolific edible fungi which offers robust flavor and nutrition. Nonetheless, this mushroom contains high moisture levels and intense respiration. Without appropriate postharvest preservation techniques, the button mushroom readily experiences browning and senescence. To ensure optimum quality, prompt cooling and appropriate storage conditions are essential. This present research investigated the postharvest quality of button mushrooms stored in a controlled atmosphere (CA) with different initial gas compositions. The findings revealed that button mushrooms in the CA group demonstrated considerable enhancements in appearance and overall quality, effectively delaying browning and senescence compared to those in the control group. The optimal gas composition is 1–3% O_2_ and 15–17% CO_2_ (CAII), which effectively inhibited the expression of polyphenol oxidase (PPO)- and lactase (LAC)-related genes in the button mushroom, maintaining a high L* value. Furthermore, the application of 1–3% O_2_ and 15–17% CO_2_ (CAII) not only preserved visual quality but also extended the postharvest shelf life of the button mushroom by minimizing metabolic activities that contribute to senescence. Moreover, 1–3% O_2_ and 15–17% CO_2_ (CAII) storage also reduced the expression levels of genes associated with ethylene synthesis, which is reflected in the gradual decrease in cell membrane permeability. Consequently, this research underscores the critical importance of controlled atmosphere storage in improving the marketability and sustainability of this widely consumed mushroom.

## 1. Introduction

*Agaricus bisporus*, commonly referred to as the button mushroom or white mushroom, stands among the most widely cultivated edible fungi worldwide with a delightful flavor and notable nutritional advantages [1,2]. However, the fruiting bodies of the button mushroom harbor high moisture content and exhibit elevated respiration rates. Consequently, they rapidly consume vital nutrients and water, which are crucial for maintaining normal physiological processes [3,4,5]. Moreover, the absence of distinct protective features on the surface of the button mushroom makes it susceptible to external physical damage and microbial infections, consequently resulting in browning and senescence [6]. To satisfy market needs, efficient postharvest storage techniques must be employed to prolong the shelf life of button mushrooms. Additionally, implementing advanced methods such as irradiation, ultrasound, essential oils, salicylic acid, citric acid, and methyl jasmonate can effectively reduce postharvest losses in fruits and vegetables [7,8,9,10,11,12]. Nevertheless, these approaches face limitations, including high expenses, safety risks, nutrient degradation, and changes in texture. Conversely, to extend the shelf life of fresh-cut fruits, vegetables, and edible fungi, researchers have studied and implemented a controlled atmosphere (CA) to inhibit microorganisms and maintain product quality. Initially, CA applications typically included reduced O_2_ and increased CO_2_ levels. This adjustment not only enhances fruit and vegetable quality but also inhibits microbial growth, contributing to a more sustainable supply chain [13]. As an illustration, CA storage significantly reduces browning in fresh in-hull walnuts and increases their postharvest lifespan by over 1.6 times [14]. Moreover, it has been proven that CO_2_-rich modified atmospheres influence energy metabolism, respiration rates, ethylene responses, and physiological changes in various fresh products during packaging and postharvest storage [15]. Research by Belay, Z. A. demonstrates that low O_2_ (1–5 kPa) and high CO_2_ (5–15 kPa) atmospheres can extend the life span of fresh-cut fruits by reducing respiration, inhibiting ethylene biosynthesis, and suppressing the growth of aerobic microorganisms [16]. Whereas, compared to horticultural products, edible fungi exhibit unique traits such as intense postharvest respiration and significant tolerance to external CO_2_ levels, suggesting that the air conditioning parameters of fresh edible mushrooms represented by the button mushroom may differ from those of fruits and vegetables. In a previous study, a short time exposure of button mushrooms to a high CO_2_ concentration resulted in reduced browning and maintained flavor quality, which means that, in contrast to the low CO_2_ atmosphere typically used in fruits and vegetables, high CO_2_ concentrations may help delay the senescence of button mushrooms and prolong their postharvest life [17]. Additionally, Amodio et al. demonstrated that a CA composition of 3% O_2_, 20% CO_2_, and 77% N_2_ effectively slows down browning and firmness loss in fresh-cut *Pleurotus eryngii* while inhibiting fungal growth [18].

Browning has the most pronounced effect on the visual appeal of edible fungi. Mechanically, browning is divided into two categories: enzymatic and non-enzymatic [19]. Enzymatic browning poses a significant challenge for horticultural products like fruits (e.g., apples, peaches, pears, bananas, and avocados) and vegetables (e.g., lettuce, potatoes, and eggplants) [20]. Recent studies indicate that the browning observed in edible fungi, particularly button mushrooms, primarily results from enzymatic processes, with polyphenol oxidase (PPO) being the chief enzyme involved [21]. In this mechanism, phenolic compounds oxidize and form quinones, which subsequently react with amino acids, leading to melanin production and resultant browning [22]. Recent findings have shown that during the storage of button mushrooms, the L* value decreases, reflecting a rise in PPO enzyme activity along with an elevated expression of the *PPO3* and *PPO4* genes [23].

Senescence is also one of the important factors that affect the shelf life of edible fungi. Research reveals that ethylene release plays a critical role in determining their storage longevity. Ethylene, identified as a plant hormone, acts as a signaling molecule that affects several metabolic pathways. As the button mushroom matures, its ethylene production may hasten senescence, resulting in changes to texture, color, and nutrient profile. Furthermore, several studies emphasize the crucial role of ethylene in the senescence of straw-rotting fungi, including the button mushroom, straw mushroom, and oyster mushroom, through the activation of genes linked to cell wall degradation, pigment accumulation, and aromatic compound production [24,25,26]. Notably, during button mushroom senescence, genes responsible for ethylene biosynthesis (*SAMS*, *ACS*, and *ACO*) play crucial roles in the postharvest management of button mushrooms.

This investigation seeks to evaluate how controlled atmosphere storage, using various initial gas compositions, impacts the visual appearance and quality characteristics of fresh button mushrooms during storage. Specifically, we focus on key indicators of freshness, conducting thorough testing and analysis on hardness, weight loss, color, and the expression of enzymatic browning-related and ethylene synthesis-related genes. Additionally, further exploration into specific storage parameters that affect browning and senescence may offer valuable insights for both consumers and producers alike.

## 2. Materials and Methods

### 2.1. Preparation of A. bisporus and Storage

The newly harvested button mushrooms were sourced from Yisheng Microbiology Co., Ltd., Lin Fen, China. On the day of harvesting, samples of button mushroom were taken to the laboratory and the experiments exclusively selected button mushrooms of superior quality, featuring sealed caps, and devoid of any imperfections. Thereafter, fresh button mushrooms were stored under each of the following conditions: (1) 1–3% O_2_ and 13–15% CO_2_ (CAI); (2) 1–3% O_2_ and 15–17% CO_2_ (CAII); (3) 3–5% O_2_ and 13–15% CO_2_ (CAIII); (4) 3–5% O_2_ and 15–17% CO_2_ (CAIV); and (5) ambient air (control). Initially, all samples underwent pre-cooling for 12 h within a cold room maintained at 4 ± 1 °C. Due to the respiration of button mushrooms affecting O_2_ and CO_2_ levels in controlled atmosphere chambers, daily adjustments were made to maintain the initial set levels of O_2_ and CO_2_ values. The samples were taken at three-day intervals. During storage, certain samples of button mushrooms were preserved for subsequent index analysis.

### 2.2. Experimental Methods

#### 2.2.1. Measurement of Weight Loss

Weight was measured on every sampling day. Three parallel experiments were conducted during each measurement, with each experimental group containing 10 mushroom samples.

#### 2.2.2. Measurement of Hardness

The texture of button mushrooms was analyzed using a TMS-PRO (FTC, CA, USA) texture analyzer. First, the stems of the button mushrooms were removed and a 2 mm thick section of the cap was cut [27]. Measurements were taken at the center of the cap and two equidistant positions on either side, making a total of 3 measurement points. The experimental conditions were as follows: testing speed: 100 mm/min, force-sensing range: 100 N, probe diameter: 0.5 cm, and distance: 8 mm. Each treatment group tested 9 mushroom samples and conducted three repeated measurements, taking the average of the results. Samples from each of the treatment groups were tested every three days.

#### 2.2.3. Measurement of Visual Appearance and Color

A digital camera (Nikon D7500, Nikon Corporation, Shanghai, China) was used to record the surface and internal appearance of button mushroom. Photos were taken of three mushrooms for each treatment every three days.

The L* value represents the brightness of button mushrooms, with higher values indicating whiter or less browning on the button mushroom surface. Changes in the color of the button mushrooms (L* value) were periodically measured using a colorimeter (WSC-S, Shanghai, China). The measurement method involved randomly selecting nine button mushrooms from each experimental group and fixing three points on the surface of the button mushroom surface. Additionally, nine button mushrooms were cut in the middle from the cap to the stem, and the color changes within the button mushrooms were measured. Measurements were taken every 3 days and three parallel experiments were conducted during each measurement, with each group containing 10 mushroom samples. The results were represented as the average.

#### 2.2.4. Measurement of Membrane Permeability

The relative membrane conductivity of the button mushroom samples was determined according to the method in accordance with Wantat, Seraypheap, and Rojsitthisak with some modifications [28]. During the experiment, 6 mushrooms were selected from each treatment group every 3 days for sampling. A perforator was employed to create openings in the mushrooms, subsequently positioning them in glass beakers filled with 20 mL of distilled water. The specimens were left undisturbed for half an hour, after which their electrical conductivity was gauged using a conductivity meter (DDS-11A, Shanghai, China), labeled P1. To eliminate mushroom tissues, the samples underwent a 10 min heating in boiling water. Following a 10 min period of cooling to ambient temperature, the electrical conductivity was reassessed, labeled as P2. The procedure was replicated three times, followed by the computation of the average. Relative membrane conductivity (%) was calculated as follows:

(1) Relative membrane conductivity (%)=(P1−P0)/(P2−P0)×100 %

where P0 represents the blank electrical conductivity (distilled water).

#### 2.2.5. Total RNA Extraction, cDNA Synthesis, and Quantitative Real-Time PCR (q-PCR)

Total RNA was extracted from the button mushrooms using Omega’s Plant RNA kit, albeit with minor alterations. The Nanodrop 2000 spectrophotometer (Thermo Scientific, Vacaville, CA, USA) was used for assessing RNA purity, followed by 1% (*w*/*v*) agarose gel electrophoresis to detect RNA degradation or contamination. The total RNA was then reverse transcribed into first-strand cDNA by using a HiScript II kit (Vazyme Biotech Co., Ltd., Nanjing, China).

Total RNA was collected and analyzed using qRT-PCR to determine the expression patterns of enzymatic browning and ethylene production genes in samples throughout storage. The four enzymatic browning-related genes and five ethylene synthesis-related genes in the button mushroom are *AbPPO1*, *AbPPO3*, *AbLAC10*, *AbLAC11*, *AbACO1*, *AbSAMS1*, *AbSAMS2*, *AbACS1*, and *AbACS2* and specific nucleotide sequence information was retrieved from the GenBank databases. The Light Cycler TM 96 System (Roche Molecular Systems, Inc., Mannheim, Germany) was utilized to conduct a quantitative real-time polymerase chain reaction. Table S1 (which is included in the Supplementary Materials) lists the primers used for EF1-α, the reference gene.

#### 2.2.6. Data Processing

The experiments underwent three rounds of analysis, and results appeared as mean ± standard deviation. Researchers employed Microsoft Excel for statistical evaluations of all experimental data, calculating standard deviations accordingly. Plots were generated using Microsoft Excel, while IBM SPSS Statistics 27 software facilitated variance analysis across all article data, utilizing Tukey’s HSD test for multiple comparisons. Statistical significance was determined at *p* < 0.05.

## 3. Results

### 3.1. Effects of CA on Visual Appearance and Quality Characteristics of A. bisporus During Storage

#### 3.1.1. Effects of CA on Browning of *A. bisporus* During Storage

**Figure 1:**
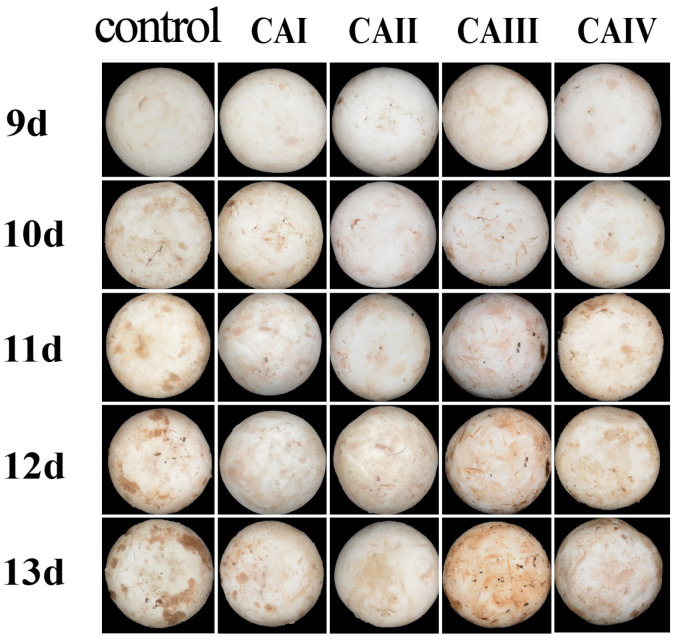
Effect of controlled atmosphere on the external appearance of *A. bisporus* during storage.

**Figure 2:**
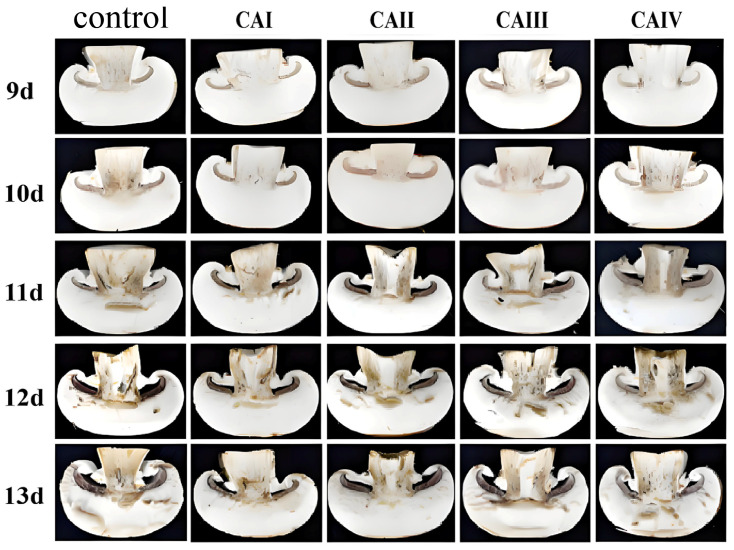
Effect of controlled atmosphere on the internal appearance of *A. bisporus* flesh during storage.

After harvesting, the external and internal color of button mushrooms in all CA groups and the control group gradually darkened with increasing storage time (Figure 1 and Figure 2). Figure 1 showed that no significant browning occurred on the button mushroom peels of the five groups within the first 10 days of storage. Notably, browning symptoms initially emerged in both control and CA samples at day 11, with the extent of browning increasing with storage duration. From day 11 onwards until the experiment concluded, the coloration of samples stored at 1–3% O_2_ and 15–17% CO_2_ (CAII) and 1–3% O_2_ and 13–15% CO_2_ (CAI) groups remained notably brighter compared to the control group. At the conclusion of the storage period, button mushrooms under 1–3% O_2_ and 15–17% CO_2_ (CAII) browned and senesced more slowly than the control group. On day 12, exterior browning occurred in the following order: cap surface, CAII < CAI < CAIV < Control < CAIII.

External browning appeared early in button mushrooms stored in both CA and control groups, while internal browning was repressed (Figure 2). Flesh browning symptoms were evident in button mushrooms kept under a controlled atmosphere and in the control after 11 days of storage, with a notable difference from controls primarily emerging between days 12 and the end of the experiment. On day 13, 1–3% O_2_ and 15–17% CO_2_ (CAII) storage retained the best color, only slightly darker than fresh button mushrooms. Button mushrooms stored at 3–5% O_2_ and 15–17% CO_2_ (CAIV group) exhibited comparable color to those kept in ambient air.

**Figure 3:**
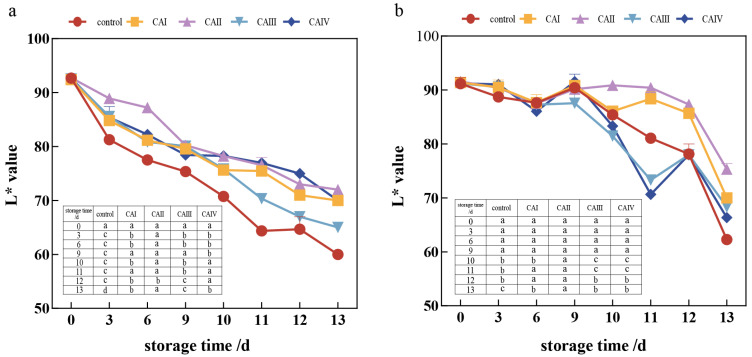
Effect of controlled atmosphere on the L* value of *A. bisporus* during storage: (**a**) surface and (**b**) flesh. The error bars represent the standard deviation (SD) for three replicates. In the table within the image, the lowercase letters a–d indicate statistically significant differences among the five groups (*p* < 0.05).

According to Figure 3a, the L* value of button mushroom caps stored in the five groups decreased over time, exhibiting rapid drops during the early and middle storage stages, with a slower reduction later. Button mushrooms maintained in a controlled atmosphere displayed a suppression of L* value reduction, particularly prominent in the middle and late phases. After storage, the L* values of button mushrooms in the 1–3% O_2_ and 13–15% CO_2_ (CAI), 1–3% O_2_ and 15–17% CO_2_ (CAII), and 3–5% O_2_ and 15–17% CO_2_ (CAIV) groups increased by 27.5%, 31.1%, and 22.4% compared to the button mushrooms in the control group, respectively. Button mushrooms in the 1–3% O_2_ and 15–17% CO_2_ (CAII) group performed better than either group, with a notable difference from controls primarily emerging between days 11 and the end of the experiment (*p* < 0.05). This result indicated that CA helped to postpone the external browning of button mushroom.

As shown in Figure 3b, the L* value of the flesh of button mushrooms stored in the five groups gradually decreased over time, with a rapid decline in the late stages. The difference between button mushrooms stored under a controlled atmosphere and controls reached statistical significance on day 10 of storage until the end of the experiment (*p* < 0.05). On day 11 and day 12, the 1–3% O_2_ and 13–15% CO_2_ (CAI) and 1–3% O_2_ and 15–17% CO_2_ (CAII) groups significantly enhanced the L* value of the flesh of button mushrooms compared to the control, 3–5% O_2_ and 13–15% CO_2_ (CAIII), and 3–5% O_2_ and 15–17% CO_2_ (CAIV) groups. At the conclusion of storage, the L* value of the flesh of button mushrooms in the control group was 62, which was 21.1%, 20.9%, 8.1%, and 16.1% lower than that of the 1–3% O_2_ and 13–15% CO_2_ (CAI), 1–3% O_2_ and 15–17% CO_2_ (CAII), 3–5% O_2_ and 13–15% CO_2_ (CAIII), and 3–5% O_2_ and 15–17% CO_2_ (CAIV) groups, respectively. Together, these results provide important insights demonstrating that the CA successfully postponed the internal browning procedure.

#### 3.1.2. Effects of CA on Weight Loss and Hardness of *A. bisporus* During Storage

**Figure 4:**
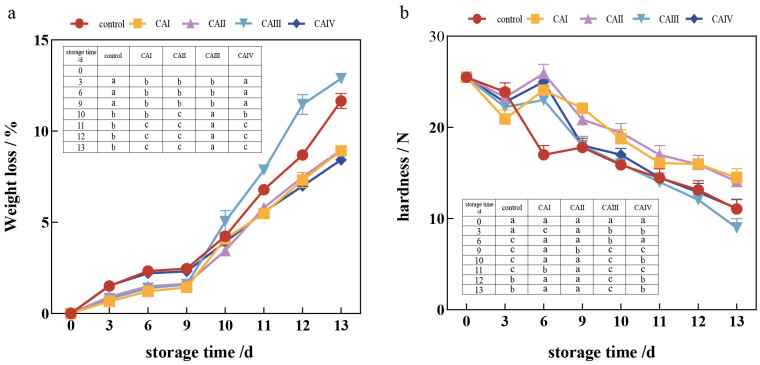
Effect of controlled atmosphere on the weight loss and hardness of *A. bisporus* during storage: (**a**) weight loss and (**b**) hardness. The error bars represent the standard deviation (SD) for three replicates. In the table within the image, the lowercase letters a–c indicate statistically significant differences among the five groups (*p* < 0.05).

As demonstrated by Figure 4a, the weight loss rate of button mushrooms stored in the five groups increased over time, with a significant increase at the final stage. On day 13 of storage, the weight loss rate of control button mushrooms rose by 34.6%, 32.7%, and 38.4% compared to button mushrooms stored in the 1–3% O_2_ and 13–15% CO_2_ (CAI), 1–3% O_2_ and 15–17% CO_2_ (CAII), and 3–5% O_2_ and 15–17% CO_2_ (CAIV) groups, respectively. The 3–5% O_2_ and 15–17% CO_2_ (CAIV) storage outperformed other groups with a significant difference observed from day 11 until the end of the investigation (*p* < 0.05). Specifically, at approximately 11–13 days of storage, the weight loss rate of button mushrooms under 3–5% O_2_ and 15–17% CO_2_ (CAIV) increased from 5.8% to 7.8%. However, the weight loss rate of samples in 3–5% O_2_ and 13–15% CO_2_ (CAIII) group increased significantly from day 10 of storage until the completion of the study, surpassing the other four groups (*p* < 0.05). This was most likely due to the low CO_2_ situation, which increased the dry-matter consumption of button mushrooms after harvesting, perhaps increasing the weight loss rate. The data presented here indicate that properly controlled atmosphere storage significantly reduces weight loss in button mushrooms.

As illustrated in Figure 4b, the hardness of the button mushroom stored in the five groups decreased over time, showing a sharp decline in the early and late storage phases, with a gradual decrease in the middle phase. On day 9, button mushrooms in the control group had a hardness of 17.6 N, which was 22.8%, 21.2%, 1.2%, and 1.1% lower than the 1–3% O_2_ and 13–15% CO_2_ (CAI), 1–3% O_2_ and 15–17% CO_2_ (CAII), 3–5% O_2_ and 13–15% CO_2_ (CAIII), and 3–5% O_2_ and 15–17% CO_2_ (CAIV) groups, respectively. At the conclusion of storage, the hardness of button mushrooms under 1–3% O_2_ and 13–15% CO_2_ (CAI), 1–3% O_2_ and 15–17% CO_2_ (CAII), 3–5% O_2_ and 13–15% CO_2_ (CAIII), and 3–5% O_2_ and 15–17% CO_2_ (CAIV) was 14.9 N, 14.2 N, 9.6 N, and 12.8 N, respectively, while the hardness of the control button mushrooms was 12.1 N. Higher carbon dioxide levels in the 1–3% O_2_ and 15–17% CO_2_ (CAII) group may reduce respiratory activity and water loss in button mushrooms, causing softening to take longer. This finding provides vital insights into higher CO_2_ concentrations proving useful in maintaining the hardness of button mushrooms during storage.

#### 3.1.3. Effects of CA on Cell Membrane Permeability of *A. bisporus* During Storage

**Figure 5:**
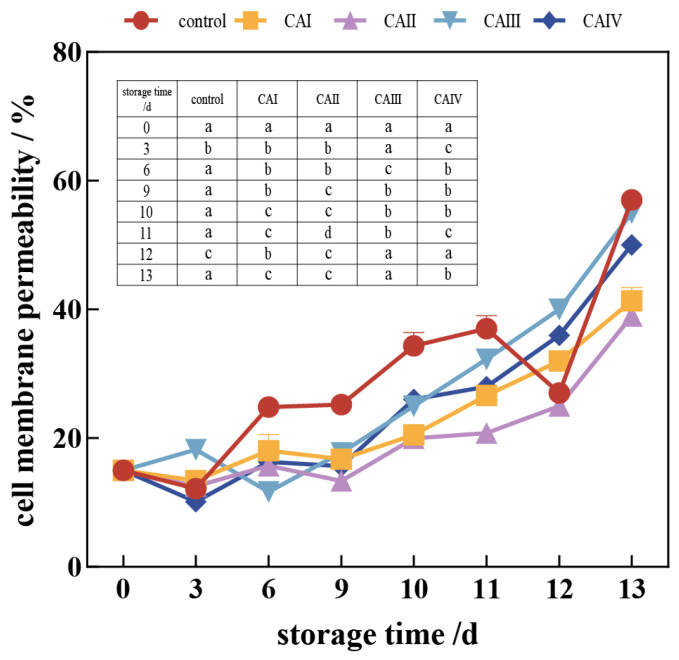
Effect of controlled atmosphere on the cell membrane permeability of *A. bisporus* during storage. The error bars represent the standard deviation (SD) for three replicates. In the table within the image, the lowercase letters a–d indicate statistically significant differences among the five groups (*p* < 0.05).

Figure 5 shows that the cell membrane permeability of the control group increased significantly with increasing storage duration, particularly after the ninth day, which could be attributed to the browning process. The permeability of cell membranes in samples stored under a controlled atmosphere was significantly lower than that of control samples, with a statistically significant difference observed from day 6 to the end of the experiment (*p* < 0.05). Specifically, control button mushrooms had 1.3, 1.4, and 1.1 times increased cell membrane permeability at the end of storage compared to button mushrooms storage under 1–3% O_2_ and 13–15% CO_2_ (CAI), 1–3% O_2_ and 15–17% CO_2_ (CAII), and 3–5% O_2_ and 15–17% CO_2_ (CAIV), respectively. This suggests that controlled atmosphere storage effectively preserves the integrity of the cell membrane, reducing the browning of tissues [29].

### 3.2. Expression Patterns of Enzymatic Browning-Related Genes

**Figure 6:**
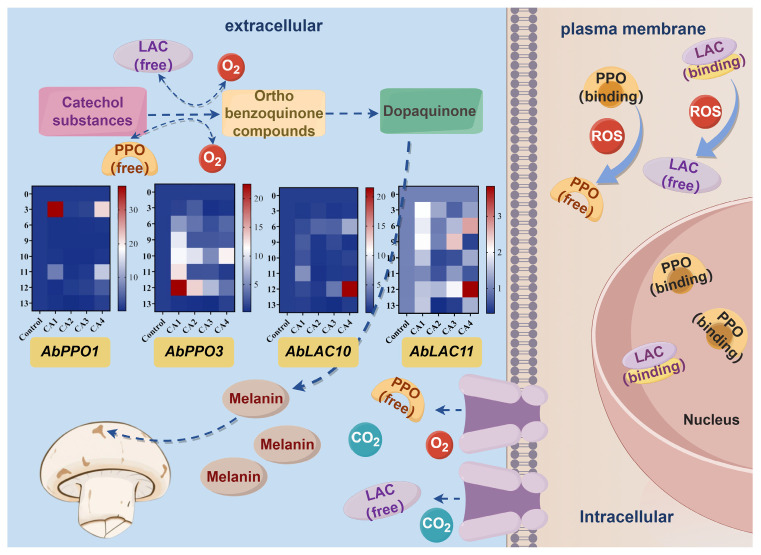
Effect of controlled atmosphere on the expression patterns of enzymatic browning-related genes of *A. bisporus* during storage (by Figdraw).

The mRNA levels of two polyphenol oxidase (PPO) and two lactase (LAC) genes in button mushrooms stored in the 1–3% O_2_ and 13–15% CO_2_ (CAI), 1–3% O_2_ and 15–17% CO_2_ (CAII), 3–5% O_2_ and 13–15% CO_2_ (CAIII), and 3–5% O_2_ and 15–17% CO_2_ (CAIV) groups for various times (0, 3, 6, 9, 10, 11, 12, and 13 days) (Figure 6) were investigated.

The 1–3% O_2_ and 15–17% CO_2_ (CAII) conditions reduced *AbPPO1* gene transcription in button mushrooms compared to the control (Figure 6). Significant differences were observed from day 6 onwards until the experiment concluded (*p* < 0.05). The 1–3% O_2_ and 13–15% CO_2_ (CAI) and 3–5% O_2_ and 13–15% CO_2_ (CAIII) storage significantly increased *AbPPO1* gene transcription in the first 3 days of storage compared to the control group (*p* < 0.05). This was most likely due to extrinsic impacts such as the removal of roots after harvesting, which might induce the expression of the *AbPPO1* gene [30]. Furthermore, after 6–13 days of storage, the transcription level of the *AbPPO1* gene reduced considerably in the 3–5% O_2_ and 13–15% CO_2_ (CAIII) group when compared to the control group (*p* < 0.05). Thus, the finding shows that *AbPPO1* was involved in the browning of the button mushroom.

The transcription levels of the *AbPPO3* gene were markedly higher in all four controlled atmosphere groups from day 6 to day 12 of storage (*p* < 0.05), which corresponded to the visual appearance (Figure 1 and Figure 2) and browning degree (Figure 3a,b). On day 13 of storage, 1–3% O_2_ and 15–17% CO_2_ (CAII) and 3–5% O_2_ and 13–15% CO_2_ (CAIII) significantly decreased *AbPPO3* transcription levels, indicating that 1–3% O_2_ and 15–17% CO_2_ (CAII) and 3–5% O_2_ and 13–15% CO_2_ (CAIII) may reduce *AbPPO3* activity in later storage stages.

The transcription patterns of the *AbLAC10* and *AbLAC11* genes are depicted in Figure 6. Throughout the storage period, the transcription levels of the *AbLAC10* and *AbLAC11* genes in all four CA groups increased initially, decreased, and then increased again. The transcription levels of *AbLAC11* and *AbLAC10* genes in button mushrooms were significantly lower under 1–3% O_2_ and 15–17% CO_2_ (CAII) when compared to the control from day 6 onwards until the experiment concluded (*p* < 0.05). Moreover, over 10–13 days of storage, the expression of *AbLAC10* and *AbLAC11* genes in CAII-stored button mushrooms increased from 0.6 to 0.9 and 0.6 to 1.4, respectively. Notably, 1–3% O_2_ and 15–17% CO_2_ (CAII) was the most effective in diminishing gene expressions of *AbLAC10* and *AbLAC11* after day 6 of storage.

### 3.3. Expression Patterns of Ethylene Synthesis-Related Genes

**Figure 7:**
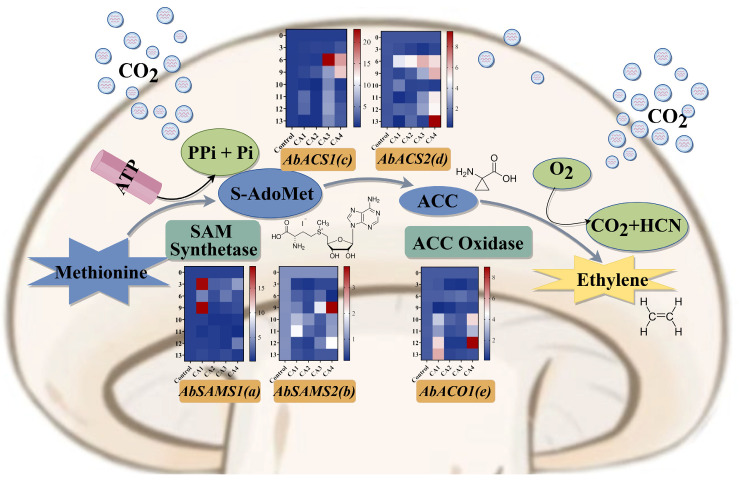
Effect of controlled atmosphere on the expression patterns of ethylene synthesis-related genes of *A. bisporus* during storage (by Figdraw): (**a**) *AbSAMS1*, (**b**) *AbSAMS2*, (**c**) *AbACS1*, (**d**) *AbACS2*, and (**e**) *AbACO1*.

The expression patterns of ethylene synthesis-related genes (*AbACO1*, *AbSAMS1*, *AbSAMS2*, *AbACS1*, and *AbACS2*) in button mushrooms stored in 1–3% O_2_ and 13–15% CO_2_ (CAI), 1–3% O_2_ and 15–17% CO_2_ (CAII), 3–5% O_2_ and 13–15% CO_2_ (CAIII), and 3–5% O_2_ and 15–17% CO_2_ (CAIV) groups for various times (0, 3, 6, 9, 10, 11, 12, and 13 days) were studied (Figure 7).

Figure 7a shows that from day 3 to day 9 of storage, the transcription level of the *AbSAMS1* gene in the 1–3% O_2_ and 13–15% CO_2_ (CAI) storage was significantly higher than that in the control group (*p* < 0.05), suggesting that this storage method may have an upregulation effect on the expression of this gene. In contrast, the expression levels under other storage conditions, such as in the 1–3% O_2_ and 15–17% CO_2_ (CAII) and C3–5% O_2_ and 13–15% CO_2_ (CAIII) groups, displayed distinct patterns throughout the storage period. The *AbSAMS1* gene expression under 1–3% O_2_ and 15–17% CO_2_ (CAII) and 3–5% O_2_ and 13–15% CO_2_ (CAIII) increased from 0.7 to 0.9, and 0.9 to 1.5 from day 6 onwards until the experiment concluded, respectively.

The transcription level of the *AbSAMS2* gene exhibited significant variations in samples stored in all four groups during storage at 4 °C (Figure 7b), peaking in the middle stages and declining towards the end. The expression level in the 3–5% O_2_ and 15–17% CO_2_ (CAIV) group surged dramatically on day 9 of storage, surpassing the other groups (*p* < 0.05). Conversely, after 10 to 13 days of storage, the transcription level of the *AbSAMS2* gene showed a marked decline in the 1–3% O_2_ and 15–17% CO_2_ (CAII) group relative to the control samples (*p* < 0.05). Furthermore, the observed reduction in transcription levels suggests that 1–3% O_2_ and 15–17% CO_2_ (CAII) can inhibit the expression of the *AbSAMS2* gene.

The transcription patterns of *AbACS1* and *AbACS2* genes were analogous in samples stored in all four groups (Figure 7c,d). The transcription of the *AbACS1* and *AbACS2* genes was significantly inhibited in the button mushrooms stored in the 1–3% O_2_ and 13–15% CO_2_ (CAI) and 1–3% O_2_ and 15–17% CO_2_ (CAII) groups (*p* < 0.05), which corresponds to the visual appearance. However, the transcription levels of the two genes increased until nine days in button mushrooms under 3–5% O_2_ and 13–15% CO_2_ (CAIII) and 3–5% O_2_ and 15–17% CO_2_ (CAIV) storage (*p* < 0.05). This was probably due to a deeper senescence of button mushrooms during later storage, which increased ethylene production and could trigger the expression of *AbACS1* and *AbACS2* genes. After 10–13 days of storage, CAII-stored button mushrooms showed an increase in *AbACS1* and *AbACS2* gene expression from 0.25 to 0.65 and 0.7 to 1.4, respectively.

The *AbACO1* gene transcription level was lower in 1–3% O_2_ and 15–17% CO_2_ (CAII) and 3–5% O_2_ and 13–15% CO_2_ (CAIII) groups compared to the control group (Figure 7e), showing a statistically significant difference from day 6 of storage through to the end of the trial (*p* < 0.05). Conversely, the transcription level of *AbACO1* was significantly heightened in 1–3% O_2_ and 13–15% CO_2_ (CAI) and 3–5% O_2_ and 15–17% CO_2_ (CAIV) groups during the later storage stages (*p* < 0.05). This suggests that the transcription of the *AbACO1* gene could be induced by external ethylene in the environment. Moreover, button mushrooms stored in the 1–3% O_2_ and 15–17% CO_2_ (CAII) group could inhibit the expression of *AbACO1*, and the inhibitory effect was particularly pronounced in the middle stage, aligning with visual assessments.

## 4. Discussion

Controlled atmosphere storage extends the storage period of fruits and vegetables by increasing the concentration of carbon dioxide (CO_2_) and/or decreasing the concentration of oxygen (O_2_) in the storage room [31]. Compared to horticultural products, edible fungi have some unique characteristics, such as high postharvest respiration and resilience to external carbon dioxide levels. Previous studies indicated that, unlike the low carbon dioxide atmospheres typically used for fruits and vegetables, high carbon dioxide levels may help slow down the senescence process of button mushrooms and extend their postharvest shelf life [18]. Furthermore, a high carbon dioxide environment impedes ethylene production and can also regulate the respiration rate and energy metabolism of button mushrooms. These conditions can synergize with low oxygen levels to manage physiological changes during the postharvest storage or packaging of various fresh products [32,33]. In the present study, observing the visual traits of button mushrooms in storage reveals that controlled atmosphere storage can reduce L* value decrease while maintaining button mushroom appearance, thus ensuring an appealing presentation for sale. Gene expression analysis indicated that 1–3% O_2_ and 15–17% CO_2_ (CAII) storage, characterized by low oxygen and high carbon dioxide levels, effectively mitigates browning and decreases the transcription levels of enzymatic browning-related genes (*AbPPO1*, *AbPPO3*, *AbLAC10*, and *AbLAC11*, respectively) in the samples.

Browning in stored button mushrooms involves an intricate chemical process, in which phenolic compounds undergo enzymatic oxidation to form quinones, which subsequently react to produce melanin [7]. This process may stem from compromised membrane integrity, as indicated by increased electrolyte leakage and malondialdehyde (MDA) accumulation [34]. Specifically, the permeability of cell membranes in samples was found to be 1.3, 1.5, and 1.1 times higher in the control than in button mushrooms stored in 1–3% O_2_ and 13–15% CO_2_ (CAI), 1–3% O_2_ and 15–17% CO_2_ (CAII), and 3–5% O_2_ and 15–17% CO_2_ (CAIV) groups, respectively, at the end of storage. This observation aligns with findings by Ayala, A [35]. Additionally, the heightened expression of the *PPO* gene and related enzyme activity may drive the oxidation of phenols, contributing to the browning of button mushrooms after harvest during storage. Furthermore, Lin et al. demonstrated that elevated CO_2_ levels inhibited browning in button mushrooms by preserving cellular integrity and inhibiting phenolic-related enzymes, such as PPO [36]. This finding aligns with the current research, showing reduced cell membrane permeability and *PPO* expression in high CO_2_ environments. Additionally, it is crucial to comprehend how environmental factors interact with gene expression to clarify the pathways involved in the oxidative stress response. Future research should focus on the impact of various gas conditions on the expression of *PPO* and *LAC* genes using both in vitro and in vivo models.

Senescence substantially affects the storage duration of button mushroom. Evidence indicates that ethylene release plays a crucial role in the maturation and senescence of bulk horticultural products, with ethylene synthesis also speeding up these processes in edible fungi. As such, understanding the mechanisms of ethylene production and its impact on button mushrooms is vital to developing appropriate storage techniques. Ethylene biosynthesis occurs when the presence of ethylene, either from internal or external sources, boosts the expression of *ACO* and *ACS* genes during the ripening of climacteric fruits [37]. Studies indicate that ethylene production in the button mushroom aligns with the ACC pathway of plants [38]. The primary mechanism involves the initial transformation of methionine into S-adenosylmethionine (SAM), facilitated by adenosine triphosphate (ATP) and SAM synthase (SAMS). Subsequently, SAM undergoes conversion to 1-aminocyclopropane-1-carboxylic acid (ACC) through the action of ACC synthase (ACS). Subsequently, ACC converts to ethylene facilitated by ACC oxidase (ACO). ACC synthase (ACS) and ACC oxidase (ACO) are the key enzymes in ethylene biosynthesis. Ethylene production correlates positively with the transcription products of the genes linked to ethylene synthesis enzymes (ACS or ACO) [39]. In tomato, the *ACS* genes *SlACS2* and *SlACS4* and *ACO* genes *SlACO1* and *SlACO4* play significant roles in ethylene synthesis [40]. Additionally, the promoters of *LeACS2* and *LeACS4* carry response elements for ethylene, wounding, and anaerobic conditions [41]. In the current work, we aimed to uncover the molecular mechanisms through which ethylene influences the postharvest maturation and senescence of button mushrooms. Based on software predictions, we identified ethylene response elements in the genes *AbACO1*, *AbSAMS1*, *AbSAMS2*, *AbACS1*, and *AbACS2* [25]. Compared to the control group, controlled atmosphere (CA) storage inhibited the expression of the *SAMS*, *ACO*, and *ACS* genes. Similar results have been reported in tomatoes [40]. Notably, 1–3% O_2_ and 15–17% CO_2_ (CAII) significantly reduced the expression of ethylene synthesis-related enzymes after 6 days of storage. The findings suggest that ethylene plays a regulatory role in the maturation and senescence of postharvest button mushrooms, resonating with climacteric fruit behavior. Furthermore, this suggests that altering the atmospheric composition could potentially influence ethylene production, subsequently affecting button mushroom growth patterns and physiological responses.

To conclude, high CO_2_ controlled atmosphere storage preserved the visual quality of button mushrooms, while inhibiting weight loss and softening. Using 1–3% O_2_ and 15–17% CO_2_ (CAII) suppressed the transcription levels of *PPO* and *LAC*, as well as ethylene synthesis-related genes, thus improving the browning and senescence of button mushrooms. These findings indicate that high CO_2_ controlled atmosphere storage has the potential to improve the nutrition, quality, and ultimately consumer preference for button mushrooms after harvesting and during storage. Additionally, tailored CA conditions for button mushrooms could be further optimized to improve their shelf life and overall quality.

## 5. Conclusions

Based on observations regarding the weight loss, L* value, hardness, cell membrane permeability, and visual appearance, CA storage maintained the storage quality of the button mushrooms over extended periods. In comparison to the other three CA groups, 1–3% O_2_ and 13–15% CO_2_ (CAI), 1–3% O_2_ and 15–17% CO_2_ (CAII), and 3–5% O_2_ and 15–17% CO_2_ (CAIV), the 1–3% O_2_ and 15–17% CO_2_ (CAII) group demonstrated the most favorable outcomes. In the meantime, the expression patterns of the enzyme-catalyzed browning-related genes *AbPPO1*, *AbLAC10*, and *AbLAC11* matched the visual appearance and browning degree, which were effectively inhibited under a high concentration of CO_2_. Additionally, results indicated that 1–3% O_2_ and 15–17% CO_2_ (CAII) lowered the expression of SAMS, ACO, and ACS enzyme-associated genes, potentially postponing the senescence of postharvest button mushrooms. What emerges from these results reported here is that high CO_2_ controlled atmosphere storage can delay button mushroom browning and ethylene synthesis, consequently increasing the postharvest shelf life of button mushrooms. Further investigations are warranted to explore the mechanisms underlying these effects, specifically focusing on the metabolic pathways influenced by elevated carbon dioxide concentrations.

## Data Availability

The original contributions presented in the study are included in the article/Supplementary Material, further inquiries can be directed to the corresponding authors.

## Supplementary Materials

The following supporting information can be downloaded at: https://www.mdpi.com/article/10.3390/foods13213486/s1, Table S1: Primer names and sequence.
