# Supplementary material for: Effect of High CO2 Controlled Atmosphere Storage on Postharvest Quality of Button Mushroom (Agaricus bisporus)
Source: Foods. 2024 Oct 30;13(21):3486. doi: 10.3390/foods13213486 (PMC11545294; doi:10.3390/foods13213486)
Supplement: Supplementary file 1 [file foods-13-03486-s001.zip › foods-3243896-supplementary.pdf]

**Table S1.** Primer names and sequence.

| Primer Names | Primer Sequences |
|---|---|
| *AbEF1α*_F | TGGTCGTGTTGAGACTGGTA |
| *AbEF1α*_R | GGGTCGTTCTTGGAATCAGA |
| *AbPPO1*-F | AAGGAGGTGGAGGTGCTG |
| *AbPPO1*-R | TATGCGCTGGTTTGGATC |
| *AbPPO3*-F | CAGCATGCGGATCACGAA |
| *AbPPO3*-R | CGACGACAAGAGACGTGAGTTC |
| *AbLAC11*-F | AGACCCAAACCAGCATCG |
| *AbLAC11*-R | CAGGGAAGTCGTACAGGAAA |
| *AbLAC10*-F | GGAATGGCAGAGGGAGAC |
| *AbLAC10*-R | TAGCCGCAACCGTTTGAT |
| *AbACO1*-F | GCATCCCTCCAGATAATGACTAA |
| *AbACO1*-R | GCTTTTCCGTATTCACCCGC |
| *AbSAMS1*-F | TTGCCAAATCTCTTGTTTCTGC |
| *AbSAMS1*-R | GTCTTCTTTCCGGTGCCGT |
| *AbSAMS2*-F | TCACATCATCCCAGATCCCAA |
| *AbSAMS2*-R | CGAGTCCACCTTCAATGCCTA |
| *AbACS1*-F | TACGGCTAACAATAACCAGGACC |
| *AbACS1*-F | AGAAGCGGGAGGATGTAAAGG |
| *AbACS2*-F | TCCCATACCGCAATACCCCCT |
| *AbACS2*-R | ACCGCATCTTACTGACCACCTT |
